# Performance Improvement of a Negative-Pressurized Isolation Room for Infection Control

**DOI:** 10.3390/healthcare9081081

**Published:** 2021-08-23

**Authors:** Fujen Wang, Citra Chaerasari, Dibakar Rakshit, Indra Permana, Kusnandar

**Affiliations:** 1 Department of Refrigeration, Air Conditioning and Energy Engineering, National Chin-Yi University of Technology, Taichung 411, Taiwan; citra.chaerasari19@gmail.com; 2 Department of Energy Science and Engineering, Indian Institute of Technology Delhi, New Delhi 110016, India; dibakar@iitd.ac.in; 3 Graduate Institute of Precision Manufacturing, National Chin-Yi University of Technology, Taichung 411, Taiwan; indra.refrigeration@gmail.com (I.P.); kusnandar11@gmail.com (K.); 4 Department of Refrigeration and Air Conditioning Engineering, Politeknik Negeri Indramayu, Indramayu 45214, Indonesia

**Keywords:** infection control, isolation room, computational fluid dynamics, ventilation performance

## Abstract

Negative-pressurized isolation rooms have been approved effectively and applied widely for infectious patients. However, the outbreak of COVID-19 has led to a huge demand for negative-pressurized isolation rooms. It is critical and essential to ensure infection control performance through best practice of ventilation systems and optimum airflow distribution within isolation rooms. This study investigates a retrofitting project of an isolation room to accommodate COVID-19 patients. The field measurement has been conducted to ensure the compliance with the design specification from the CDC of Taiwan. The pressure differentials between negative-pressurized isolation rooms and corridor areas should be at least 8 Pa, while the air change rate per hour (ACH) should be 8–12 times. Computational fluid dynamics (CFD) is applied to evaluate the ventilation performance and contamination control. Different layout arrangements of exhaust air have been proposed to enhance the ventilation performance for infection control. A simple projected air-jet curtain has been proposed in the simulation model to enhance extra protection of medical staff. The resulting ventilation control revealed that the contamination control can be improved through the minor adjustment of exhaust air arrangement and the application of an air-jet curtain.

## 1. Introduction

The outbreak of COVID-19, caused by the novel SARS-CoV-2, spread rapidly worldwide in early 2020. The transmission of SARS-CoV-2 was evaluated through experimental study: it was plausible that the spread can be generated through respiratory droplets and possibly small aerosols [1]. The dispersion of droplets and aerosols has considerable relevance in the spread of this virus, often present in small particles with a diameter of less than 5 μm [2]. The airborne transmission risk of COVID-19 was also confirmed by the Center for Disease Control and Prevention (CDC), which advises that the droplets could spread through inhalation or ingestion by susceptible persons [3]. The possibility of human-to-human transmission may occur through respiratory droplets by coughing, sneezing, talking and direct contact with an infected person [4]. The emergence of these pathogens addresses new concerns on infection control, which elevates the risk of transmission from patients to medical staff. Furthermore, the outbreak resulted in high demand for negative-pressurized isolation rooms to contain emitted pathogens from contagious patients in order to control the transmission [5]. The ANSI/ASHRAE Standard-170 provides a general guideline on the ventilation of health care facilities, which includes isolation rooms [6]. Specific requirements for isolation rooms such as pressurization, ventilation, and filtration using HEPA filters are needed to dilute and remove infectious airborne contagions. The CDC recommends that the level of negative pressure achieved depends on the difference in the ventilation exhaust and supply air by supplying less air to the area than is exhausted from it, which should be around 10% larger than supply air volume [7]. Numerous international and professional organizations publish guidelines and recommendations for isolation rooms; therefore, the standard guidelines are different in every country. In this case, the requirements of isolation rooms in Taiwan should have the air change rate per hour (ACH) between 8–12 ACH and the differential pressure with its adjacent space should be at least 8 Pa [8,9]. Moreover, heating, ventilation, and air-conditioning (HVAC) systems play a significant role in increasing or reducing the spreading of infection in isolation rooms. Efficient ventilation system design could help mitigate the spread of infectious particulates and reduce the transmission of disease [10].

Computational fluid dynamics (CFD) simulation is employed to investigate the airflow pattern, pressurization, and control contamination in isolation rooms. Thus, it helps to find a better design layout for supply and exhaust air, in order to improve the performance of ventilation and achieve contamination control. A number of experimental studies have been conducted through CFD numerical analysis related to negative-pressurized isolation rooms. A recent study by Boro et al. [11] on the role of HVAC in the waiting room and patient room of a children’s hospital focused on the diffusion of SARS-CoV-2. Through CFD simulation, it revealed that higher airflow would reduce the contaminant concentration, yet it also leads to a substantial increase in turbulent air motions and spreading the droplets and aerosol throughout the room. An innovative local exhaust ventilation system could significantly reduce droplets and contaminated air in the children’s rooms. Moreover, exhaust vents located behind bed heads would provide effective infection control, as found by Kao and Yang [12]. In addition, an experimental and simulation study has been conducted by Cho [13] on the placement of two wall-mounted exhaust air grilles beside the patient’s head. The result shows it could provide a ready flow path for the airborne contaminant to directly exit the isolation room. Another CFD simulation study on the airflow patterns and thermal comfort based on different locations of supply diffusers and exhaust air grilles has been conducted by Khankari [14]. The study demonstrates that the location of supply air across the patient and exhaust grille over patients’ heads can work collaboratively to establish effective contaminant control.

Wang and Huang [15] investigated the airflow distribution and concentration decay of a negative-pressurized biosafety laboratory. Through CFD simulation, it was revealed that ventilation performance can be improved just by relocating the supply air with a high efficiency particulate air filter (HEPA). Other research revealed that the arrangement could help the control of bacteria’s concentration in the isolation room [16]. A comprehensive review on the distribution patterns and air movement for infection control concerns has been presented by Pereira and Tribess [17]. Their study identified control strategies that could reduce the risks of airborne contamination in surgery rooms. Another study by Cao et al. [18] based on a new ventilation concept, called protected occupied zone ventilation (POV), has been investigated to improve the ventilation quality and to prevent cross contamination by using a downward plane air-jet. Besides that, the angle of the air curtain could influence the effectiveness in protecting healthcare personnel from the infected patient. By applying an angle of 0–40°, it was found to be effective in confining cross-infection risk [19,20].

In this study, field measurement was conducted in a retrofitted negative-pressurized isolation room located within a hospital in Taiwan. The field measurement data were used for boundary conditions to conduct the CFD simulations. The simulation results have been investigated extensively on the airflow patterns and contamination concentrations to improve the ventilation performance and contaminant control. There are two strategies proposed in this simulation; the first one is the arrangement of exhaust air grilles to conduct a better performance of the ventilation system. The second one is to conduct an additional air-jet curtain placed between the patient’s bed and the medical staff in order to improve the contaminant removal. Different air-jet speeds were evaluated to find the best performance on contaminant control.

## 2. Negative-Pressurized Isolation Room 

### 2.1. System Description

**Figure 1:**
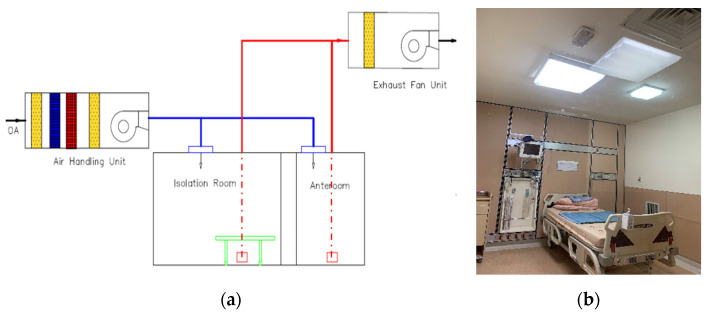
The investigated isolation room: (**a**) HVAC system; (**b**) snapshot.

The schematic diagram of the investigated negative-pressurized isolation room is shown in Figure 1. An Air Handling Unit (AHU) is used to distribute the air into the isolation room as shown in Figure 1a. The snapshot of the isolation room is shown in Figure 1b. Supply air will be delivered to the room through a square air diffuser with dimensions of 600 × 600 (mm) mounted on the ceiling. The exhaust air will be removed through a wall-mounted exhaust grille with dimension of 300 × 300 (mm) right beside patient’s bed. To establish negative pressure in the isolation room, the exhaust flow rate has been modulated by an inverter with variable air volume control depending on pressurization specification.

The negative-pressurized isolation room design conditions need to comply with standard guidelines from the CDC of Taiwan. The existing condition of the isolation room should have an air change rate of 12 ACH while maintaining the differential pressure at 8 Pa. Through field measurement, the airflow rate is measured at the outlet of a supply diffuser. An airflow capture hood meter is used to enclose the air supply diffuser, which has an airflow rate of 440 m^3^/h. The air change rate per hour inside the isolation room could be found from the rate of supply airflow divided by the room’s dimensions, so the air change rate per hour is at 15 ACH. The other specified design condition is that the temperature of the isolation room will be maintained at 22 ± 2 °C. Measurement data would be used as the inputs for all boundary conditions in the numerical simulation.

### 2.2. CFD Simulation and Improvement Strategy

**Figure 2:**
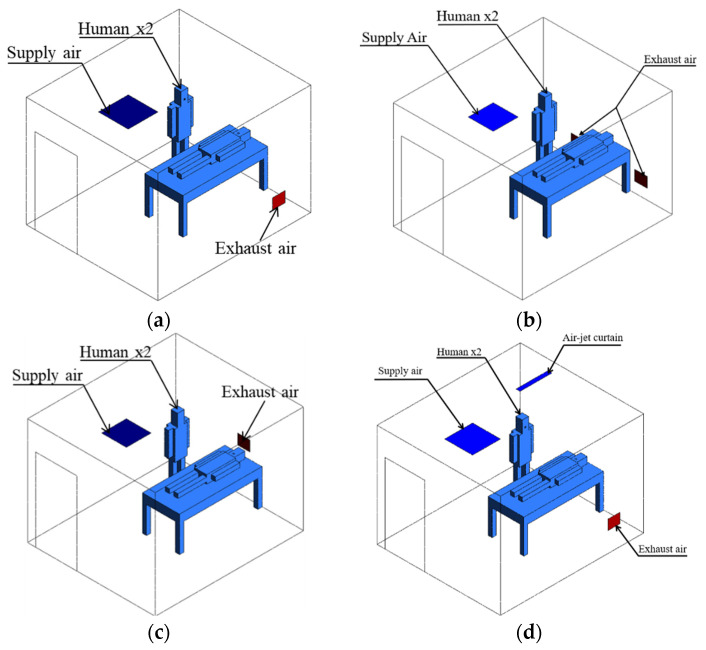
The geometry model of the isolation room based on ventilation strategies: (**a**) case 1 (existing model); (**b**) case 2 (two exhaust air grilles); (**c**) case 3 (exhaust air grille above head); (**d**) case 4 (case 1 with air-jet curtain).

The simulation of the negative-pressurized isolation room was performed by using a commercially available code Ansys fluent [21]. The dimensions of length, width, and height, respectively, for the isolation room are 3.6 × 3.1 × 2.6 (m). The 3D geometry of the isolation room is made based on the existing layout as shown in Figure 2a. This ventilation strategy may not efficiently reduce the contaminant concentration of an infectious source due to the location of exhaust air. Three cases were analyzed in this study based on the location of exhaust air to find the best arrangement. Furthermore, there will be an additional proposed case to improve the ventilation system by implementing an air-jet curtain to protect the medical staff from the patient’s contamination. The dimension of air-jet curtain for width and length, respectively, are 0.1 m and 0.8 m. Various cases based on locations of exhaust air grilles are described below.

- Case 1: Ceiling supply air passes across the patient, and right wall-mounted exhaust air grilles are located about 40 mm above the floor. This is the base existing model for the negative-pressurized isolation model (Figure 2a).
- Case 2: Two wall-mounted exhaust air grilles are located beside the patient’s head at 25 mm above the floor (Figure 2b).
- Case 3: Wall-mounted exhaust air grilles are located behind the patient’s head at 1000 mm above the floor (Figure 2c).
- Case 4: Case 1 with an additional ceiling-mounted air-jet curtain, which is placed between the patient’s bed and the medical staff (Figure 2d).

### 2.3. Boundary Conditions and Initial Conditions

Monitoring carbon dioxide (CO_2_) as a contamination source concentration exhaled by patients can provide valuable assessment to ensure adequate ventilation for patients [22]. Contaminants such as CO_2_ and other waste anesthetic gases can cause severe complications for patients if they are not handled properly; elevated CO_2_ concentrations can be related to an increase in other indoor contaminant concentrations. Thus, the concentration of CO_2_ generated inside the isolation room can be considered as a good indicator of the ventilation efficiency in the process of air renewal [23]. The CO_2_ concentration in the outdoor atmosphere is about 400 ppm, which will be the concentration value for the supply HEPA CO_2_ [24]. The initial condition CO_2_ concentration in the isolation room is assumed at 1000 ppm.

Transient numerical simulations were implemented to determine concentration level and were recorded over a time period of 900 s. The boundary conditions of numerical simulation are shown in Table 1. The patient is assumed to exhale air with a concentration around 38,000 ppm [25]. Heat flux generated from each person is at 46.52 W/m^2^ [25]. The walls and door were assumed to have convection heat transfer from ambient temperature with a heat transfer coefficient of 14.7 W/m^2^ K and 0.9 W/m^2^ K, respectively. CFD simulation would numerically solve a set of equations for the conservation of mass, momentum, energy, and turbulence quantities [26]. The realizable k-ε was selected as the turbulence model for its accurate prediction and extensive use in the research of airflow modeling. Pressure-implicit with splitting of operators (PISO) was used as the pressure-velocity coupling equations.

### 2.4. Grid Independence Test

**Figure 3:**
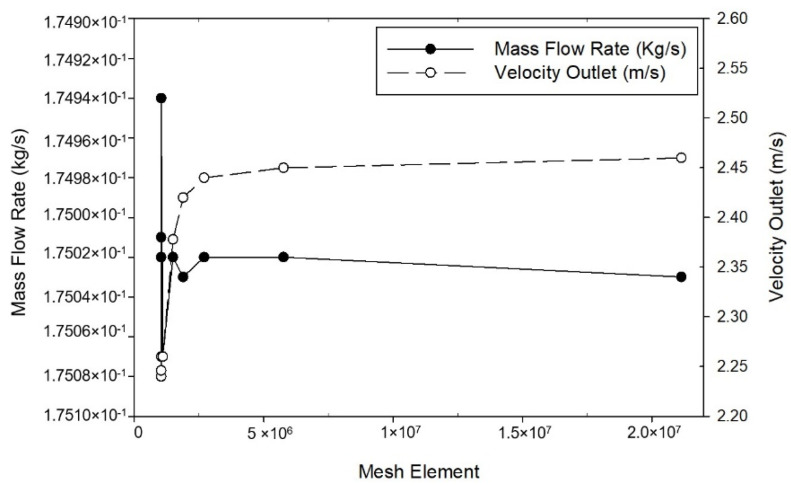
Grid independence test based on mass flow rate and velocity outlet.

The finite control volume method divides the computational domain into small cells that have a tetrahedral grid. The mesh topology was determined by refining the mesh until grid independence of the mass flow rate was achieved. The mass flow rate and velocity outlet differences are insignificant between the grids containing 1,490,698 cells and for the next range of grids as shown in Figure 3. The grid independence test proves the numerical robustness of CFD simulation in solving this problem and a grid containing 1,490,698 cells is employed for the simulation.

## 3. Results and Discussion

### 3.1. Arrangement of Exhaust Air Grilles

**Figure 4:**
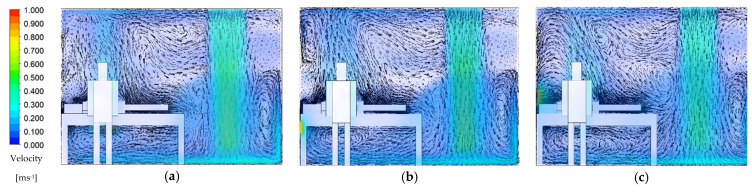
Airflow distribution pattern at 480 s: (**a**) case 1 (existing model); (**b**) case 2; (**c**) case 3.

Transient numerical simulation was conducted for a 900 s duration by assuming the initial condition of CO_2_ concentration level at 1000 ppm, which then would dilute to a background concentration level of 400 ppm. The average CO_2_ concentration level, temperature, pressure, and air velocity inside the isolation room were chosen to evaluate the performance of the ventilation system and contaminant removal. The results of the airflow distribution pattern from transient simulation over 480 s for each case are presented in the form of color contours and vector plots, as shown in Figure 4. Plane y-z at 2.85 m (cross-section right at the patient’s mouth area) was chosen for this demonstration. 

The laminar airflow distribution extends downward after leaving the supply air before it spreads out thoroughly across the isolation room, which is beneficial for infection control purposes. The location of exhaust air for each case can be determined from the higher contours formed around them. In every case, the airflow forms a recirculating pattern under the bed and in front of the entrance door. Some air will flow over the patient’s body and become entrained back into the supply airstream. In case 1 (Figure 4a), exhaled air released from the patient will hit the wall behind then become entrained back into the supply airstream. Eventually, the contaminated air from the patient can spread into the room along with the airstream supply before leaving into the wall-mounted exhaust air. The exhaled airflow for case 2 is similar to the previous case, as displayed in Figure 4b. Exhaled air forms a recirculating pattern near the ceiling before becoming entrained back into the supply airstream. This arrangement for the exhaust air has little effect on removing exhaled air by the patient even though it was placed beside the patient’s head. Case 3 has the best results in removing exhaled air by the patient, as shown in Figure 4c. The exhaust air grille placed right behind the patient’s head facilitates an easy passage: this configuration can provide a direct path for airborne contaminants exhaled from the patient without leading to recirculation air back into supply airstream. This revealed that the ventilation performance can be improved by relocating the exhaust air. 

**Figure 5:**
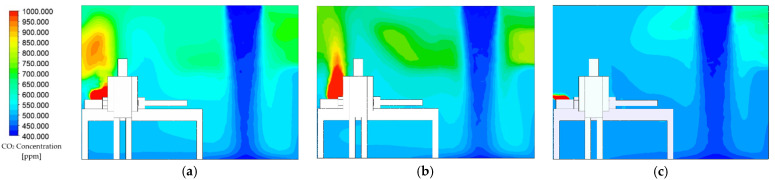
Concentration distribution contour at 480 s: (**a**) case 1 (existing model); (**b**) case 2; (**c**) case 3.

Figure 5 shows concentration distribution contours at 480 s for each case. In every case, a higher concentration can be observed around the upper right wall region because of the formation of stagnant air was formed in this region. In case 1, as shown in Figure 5a, the higher CO_2_ concentration will be accumulated above the patient’s head. The higher concentration region indicates the probable exhaled air pathway by the patient. Case 2, as shown in Figure 5b, revealed the region that formed higher CO_2_ concentrations that were more intense than case 1. Such relocation of the exhaust air grille did not significantly improve the ventilation performance. Concentration distribution shows the optimized results in case 3 (shown in Figure 5c). Higher concentrations only form around the patient’s mouth region, from which it will then directly exit through the exhaust air grille placed behind the patient’s head. It indicates that there will not be recirculation of exhaled air from the patient into the supply airstream. This arrangement can help reduce the CO_2_ concentration within the isolation room. The concentration contour in each case of the ventilation system can represent the exposure level of airborne contamination to the medical staff. Case 3 has a better ventilation performance based on the airflow pattern and contaminant concentration than other cases. Cases 1 and 2 have almost similar results on the airflow pattern and the contaminant concentration. 

**Figure 6:**
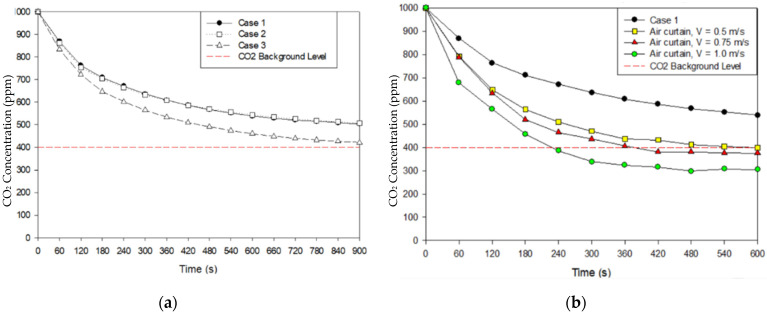
Concentration decay: (**a**) various cases based on locations of exhaust air; (**b**) case 1 with air-jet curtain.

Based on the numerical simulation results, it can be observed that the original case (case 1) is still feasible in diluting the CO_2_ concentration as shown in Figure 6. The average CO_2_ concentration was observed on plane x-z at 1.2 m height, which is the likely height of exhaled air from the patient and the most likely level of contaminant of the medical staff. Over a 900 s duration, the CO_2_ concentration level can be reduced to 504 ppm for case 1 and 508 ppm for case 2. Even though two wall-mounted exhaust air grilles have been arranged right beside the patient’s head, it shows that case 1 has lower performance in removing the contaminant. It may occur due to the medical staff positioned right in front of exhaust air grilles area, which can cause an obstruction in removing the airborne contaminant. As for case 3, the results show better performance than the others, which can reduce CO_2_ concentration to 422 ppm.

**Figure 7:**
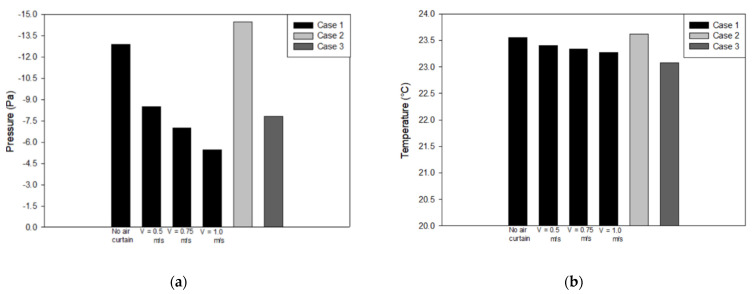
Various cases based on locations of exhaust air: (**a**) pressure; (**b**) temperature.

Figure 7 shows the average result for pressurization and temperature distribution on plane x-z at 1.2 m height. The improvement case was observed extensively with the arrangement of the exhaust air and the application of air-jet curtains with different face velocities. Figure 7a shows that case 1 and case 2 have complied with the standard for negative-pressurized isolation rooms at −12.89 Pa and −14.48 Pa, respectively. Case 3 has not complied with the specification pressure design at −7.8 Pa. As mentioned above, the standard differential pressure for negative isolation rooms should be maintained at least to 8 Pa. Figure 7b revealed the temperature distribution average for each case has almost similar results with a range of 23.0 °C to 23.6 °C.

### 3.2. Air-Jet Curtain

In order to protect medical staff from the exposure of patients’ contaminants, another strategy has been explored in this study. The results from the rearrangement of the exhaust air grilles show the original case is still feasible in reducing the contaminant and can maintain the negative pressure, but it would need to be supplemented with an additional feature, namely an air-jet curtain, to improve the ventilation system.

The velocity of the air-jet curtain was observed with different face velocities at 0.5 m/s, 0.75 m/s, and 1.0 m/s, respectively. Transient numerical simulation was conducted for 600 s duration with the same initial condition as the previous strategy. The average concentration decay was observed on plane x-z at 1.2 m, as shown in Figure 6b. It reveals that the concentration level can be diluted to the background level standard below 400 ppm within 600 s simulation. The results also reveal that the higher the velocity of the air-jet curtain, the quicker the concentration decay will reduce. However, it will have an effect on the pressurization inside isolation room as shown in Figure 7a. The higher velocity of the air-jet curtain will cause the negative pressure to decrease as well until it cannot maintain the differential pressure at −8 Pa. Figure 7b shows the temperature results for the improved ventilation system with the additional air-jet curtain. It indicates that the location of exhaust air in case 1 can be improved extensively with an additional air-jet curtain.

### 3.3. Cough Model

**Figure 8:**
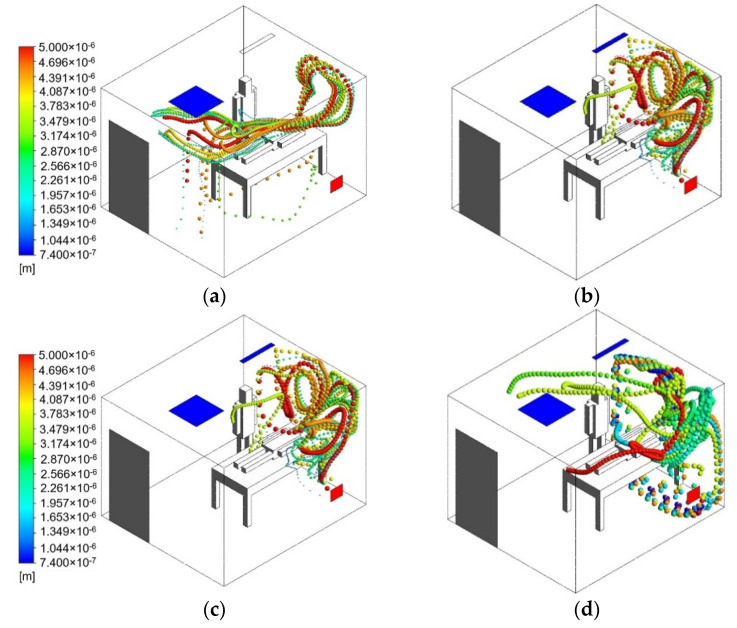
Particle trajectory pathway from the patient (0–120 s): (**a**) without air curtain (**b**) V = 0.5 m/s; (**c**) V = 0.75 m/s; (**d**) V = 1.0 m/s.

Exposure of infectious aerosols that have been emitted to the isolation room by the patient can cause a harmful transmission during the movement of supply air to the exhaust. Recent studies have shown that, in some cases, when the supply flow rate increases, the generated airflow patterns will cause increased exposure to the infectious aerosols aerosolized by breathing or coughing [27]. Based on another study, coughing generates droplets that travel between 6 and 28 m/s and range between 50 μm and 400 μm in diameter [28,29,30]. In order to find the effective velocity for the usage of an air-jet curtain, an aerosol model has been analyzed with the motion of particles exhaled by a coughing patient. The effect of the particle spread coming from a coughing patient was modeled using discrete phase model (DPM). A transient numerical simulation was conducted for 120 s duration, in this case the coughing model will start at the 5th s and will end at the 7th s. A velocity of 10 m/s and a diameter distribution of 50 μm and 400 μm were used to define the initial condition of cough droplets in this simulation. The results of the particle trajectory pathway from the patient for the transient simulation is shown in Figure 8. The particles emitted by the coughing patient will be entrained by the main supply airstream and air-jet curtain, and aerosol particles from the cough droplets will be dispersed inside the isolation room before leaving through exhaust air. 

Figure 8a represents the original case 1 without an additional air-jet curtain. It can be observed from 0 s until 120 s that medical staff could be exposed to aerosol particles emitted from coughing patients. The particle trajectory pathway was carried by the supply airstream behind the medical staff and spread out, then eventually leaving through the exhaust air. It can be observed that this configuration did not effectively decrease the aerosol particles. The results of the particle probable trajectory pathway from the coughing patient with an additional air-jet curtain are shown in Figure 8b–d. The higher velocity of the air-jet curtain will decrease particle residence time and distance traveled. However, increasing the velocity above 0.75 m/s does not provide any additional benefit in terms of reducing personal exposure. The air-jet curtain with a velocity of 1 m/s did not provide a laminar airflow; instead, it created a turbulent air pattern. Turbulent airflow could suspend infectious particles within breathing zones. Figure 8d shows that the particle trajectory pathway can penetrate through the air-jet curtain and transmit to the medical staff. 

**Figure 9:**
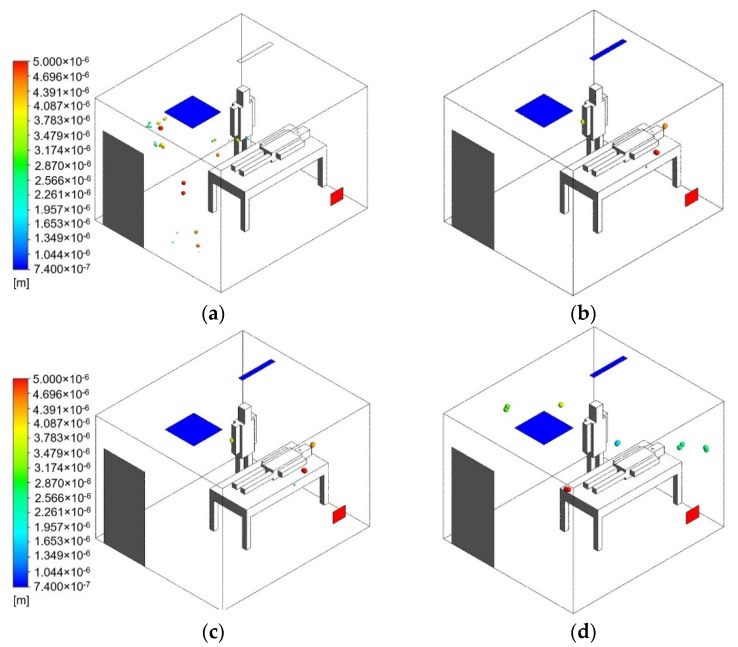
Particle trajectory pathway from the patient (at 120 s): (**a**) without air curtain (**b**) V = 0.5 m/s; (**c**) V = 0.75 m/s; (**d**) V = 1.0 m/s.

The final results of particles without the air-jet curtain at 120 s can be observed in Figure 9a. Larger particles are either deposited in the exhaust air or fall to the ground by gravity. Meanwhile, the medium- to small-sized particles are still entrained in the supply airstream. The final results of particles at 120 s with an additional air-jet curtain can be seen in Figure 9b–d. This approach shows that adding an air-jet curtain and increasing its velocity creates a higher level of dilution and reduces exposure to infectious aerosol.

## 4. Conclusions

In this study, the transient simulation of a negative-pressurized isolation room has been carried out to evaluate the best layouts for ventilation system arrangement and contamination control. The results revealed that the ventilation performance can be improved by the rearrangement of the exhaust air grille’s location. The exhaust air located near the patient’s head reveals better ventilation performance. However, the original case can still be optimized by using an air-jet curtain to improve the contaminant control inside the isolation room. It also revealed that the higher velocity of air-jet will induce faster concentration decay. A velocity of 0.5 m/s for an air-jet curtain is found to be the most effective in diluting the contamination to the background level below 400 ppm and in reducing exposure of aerosol particles from the coughing patient. It can maintain the specification of the pressure differential at 8 Pa within the isolation room. It is also expected that the application of an air-jet curtain will enhance the protection for medical staff in the isolation room.

## Figures and Tables

**Table 1:** Boundary conditions for numerical simulation.

Parameters	Supply Air	Exhaust Air	Exhaled Air by Patient
Velocity (m/s)	0.3	-	0.18
Temperature (°C)	22	24	37
CO_2_ Concentration (ppm)	400	-	38,000
Pressure (Pa)	-	−15	-

## Data Availability

The data presented in this study are available on request from the corresponding author.
